# Causal association of plasma circulating metabolites with nephritis: a Mendelian randomization study

**DOI:** 10.3389/fnut.2024.1364841

**Published:** 2024-05-03

**Authors:** Fengling Shao, Yingling Yao, Dunchu Weng, Runzhi Wang, Ruiling Liu, Yongjia Zhang, Erhan Li, Mengdi Wang, Yuewu Tang, Yubin Ding, Yajun Xie

**Affiliations:** ^1^The Ministry of Education, Key Laboratory of Laboratory Medical Diagnostics, The College of Laboratory Medicine, Chongqing Medical University, Chongqing, China; ^2^Department of Obstetrics and Gynecology, Women and Children’s Hospital of Chongqing Medical University, Chongqing, China; ^3^Joint International Research Laboratory of Reproduction and Development of the Ministry of Education of China, School of Public Health, Chongqing Medical University, Chongqing, China; ^4^Department of Pharmacology, Academician Workstation, Changsha Medical University, Changsha, China; ^5^Department of Nephrology, Chongqing Three Gorges Central Hospital, Chongqing University Three Gorges Hospital, Chongqing, China

**Keywords:** plasma circulating metabolites, nephritis, two-sample Mendelian randomization, therapeutic agents, bioinformatics

## Abstract

**Background:**

Nephritis is a pivotal catalyst in chronic kidney disease (CKD) progression. Although epidemiological studies have explored the impact of plasma circulating metabolites and drugs on nephritis, few have harnessed genetic methodologies to establish causal relationships.

**Methods:**

Through Mendelian randomization (MR) in two substantial cohorts, spanning large sample sizes, we evaluated over 100 plasma circulating metabolites and 263 drugs to discern their causal effects on nephritis risk. The primary analytical tool was the inverse variance weighted (IVW) analysis. Our bioinformatic scrutiny of GSE115857 (IgA nephropathy, 86 samples) and GSE72326 (lupus nephritis, 238 samples) unveiled anomalies in lipid metabolism and immunological characteristics in nephritis. Thorough sensitivity analyses (MR-Egger, MR-PRESSO, leave-one-out analysis) were undertaken to verify the instrumental variables’ (IVs) assumptions.

**Results:**

Unique lipoprotein-related molecules established causal links with diverse nephritis subtypes. Notably, docosahexaenoic acid (DHA) emerged as a protective factor for acute tubulointerstitial nephritis (ATIN) (OR1 = 0.84, [95% CI 0.78–0.90], p1 = 0.013; OR2 = 0.89, [95% CI 0.82–0.97], p2 = 0.007). Conversely, multivitamin supplementation minus minerals notably increased the risk of ATIN (OR = 31.25, [95% CI 9.23–105.85], *p* = 0.004). Reduced α-linolenic acid (ALA) levels due to lipid-lowering drugs were linked to both ATIN (OR = 4.88, [95% CI 3.52–6.77], *p* < 0.001) and tubulointerstitial nephritis (TIN) (OR = 7.52, [95% CI 2.78–20.30], *p* = 0.042). While the non-renal drug indivina showed promise for TIN treatment, the use of digoxin, hydroxocobalamin, and liothyronine elevated the risk of chronic tubulointerstitial nephritis (CTIN). Transcriptome analysis affirmed that anomalous lipid metabolism and immune infiltration are characteristic of IgA nephropathy and lupus nephritis. The robustness of these causal links was reinforced by sensitivity analyses and leave-one-out tests, indicating no signs of pleiotropy.

**Conclusion:**

Dyslipidemia significantly contributes to nephritis development. Strategies aimed at reducing plasma low-density lipoprotein levels or ALA supplementation may enhance the efficacy of existing lipid-lowering drug regimens for nephritis treatment. Renal functional status should also be judiciously considered with regard to the use of nonrenal medications.

## 1 Introduction

Nephritis, a prevalent renal disorder, is pathologically categorized into glomerulonephritis and tubulointerstitial nephritis, among other types (1, 2). It is further classified by etiology, including drug-induced nephritis, IgA nephropathy, lupus nephritis, and more (3–5). With the global prevalence of chronic kidney disease (CKD) due to nephritis exceeding 10% (6), this condition poses a substantial and growing public health concern worldwide. Thus, there’s an escalating need for early identification and mitigation of risk factors within high-risk populations, essential for alleviating the impact on global public health.

In recent years, research has unveiled abnormal plasma metabolite levels in nephritis patients, notably associated with lipoprotein metabolism (7). Disruptions in lipid metabolism are recognized as a contributing factor in nephritis and its progression to chronic kidney disease (CKD) (8, 9). Specifically, variants in the apolipoprotein L1 (APOL1) gene have been linked to APOL1 nephropathy (10). Notably, drugs tested in randomized controlled trials for nephritis primarily target immune and inflammatory pathways rather than lipid metabolism (11–14), often due to trial complexities, expenses, and the prolonged nature of nephritis (15–17). The causative link between plasma metabolites and nephritis risk, alongside the effectiveness of lipid-lowering drugs in treatment, remains uncertain.

Genome-wide association studies (GWAS) and Mendelian randomization (MR) stand as formidable tools in drug development and assessing disease risk (18, 19). GWAS offers cost-effective genetic data, and MR harnesses this information for causal inferences, mitigating environmental confounding to unveil genetic-disease associations (20, 21). Often called “nature’s randomized controlled trials,” MR investigates whether carriers of risk alleles exhibit distinct disease risks, delving into causal connections between risk factors, drug targets, and eventual outcomes (22–24).

In this investigation, we employed Mendelian randomization (MR) analysis to examine potential causal links between plasma metabolites, particularly lipoproteins, lipid-lowering medications, and nephritis. Utilizing data from an extensive genome-wide association study (GWAS) encompassing hundreds of thousands of samples, we uncovered connections between circulating metabolites and the risk of nephritis. Remarkably, diverse types of nephritis displayed unique associations with specific circulating metabolites. Moreover, across two distinct European cohorts, we identified DHA as a risk factor for acute tubulointerstitial nephritis (ATIN). Our analysis also delved into the influence of lipid-lowering drugs on nephritis outcomes and probed the viability of drugs commonly used in other conditions for potential nephritis treatment. Further validation using transcriptome data underscored the pivotal role of lipid metabolism in nephritis pathogenesis.

## 2 Method

### 2.1 Data sources

**Figure 1:**
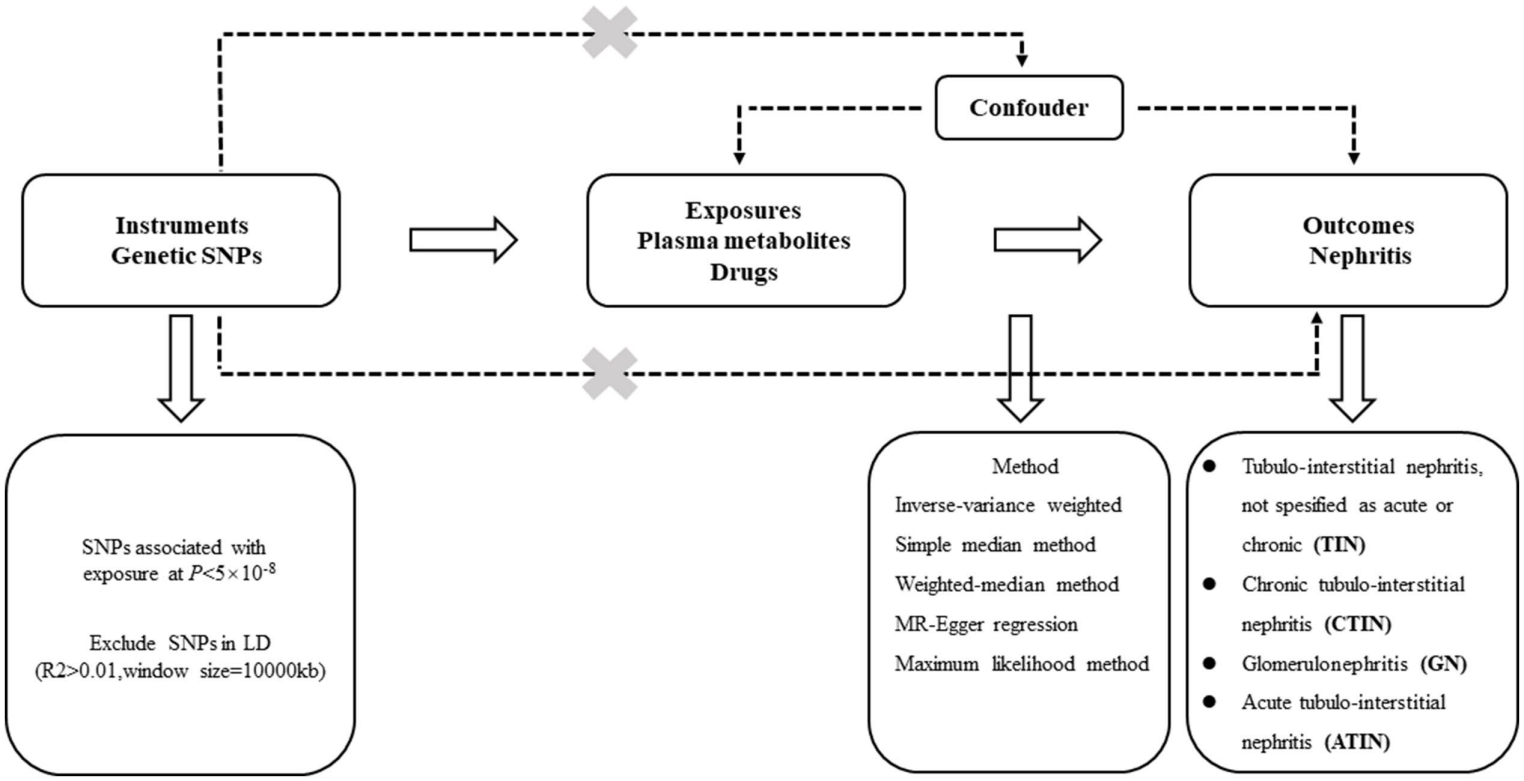
Directed acyclic graph.

**Figure 2:**
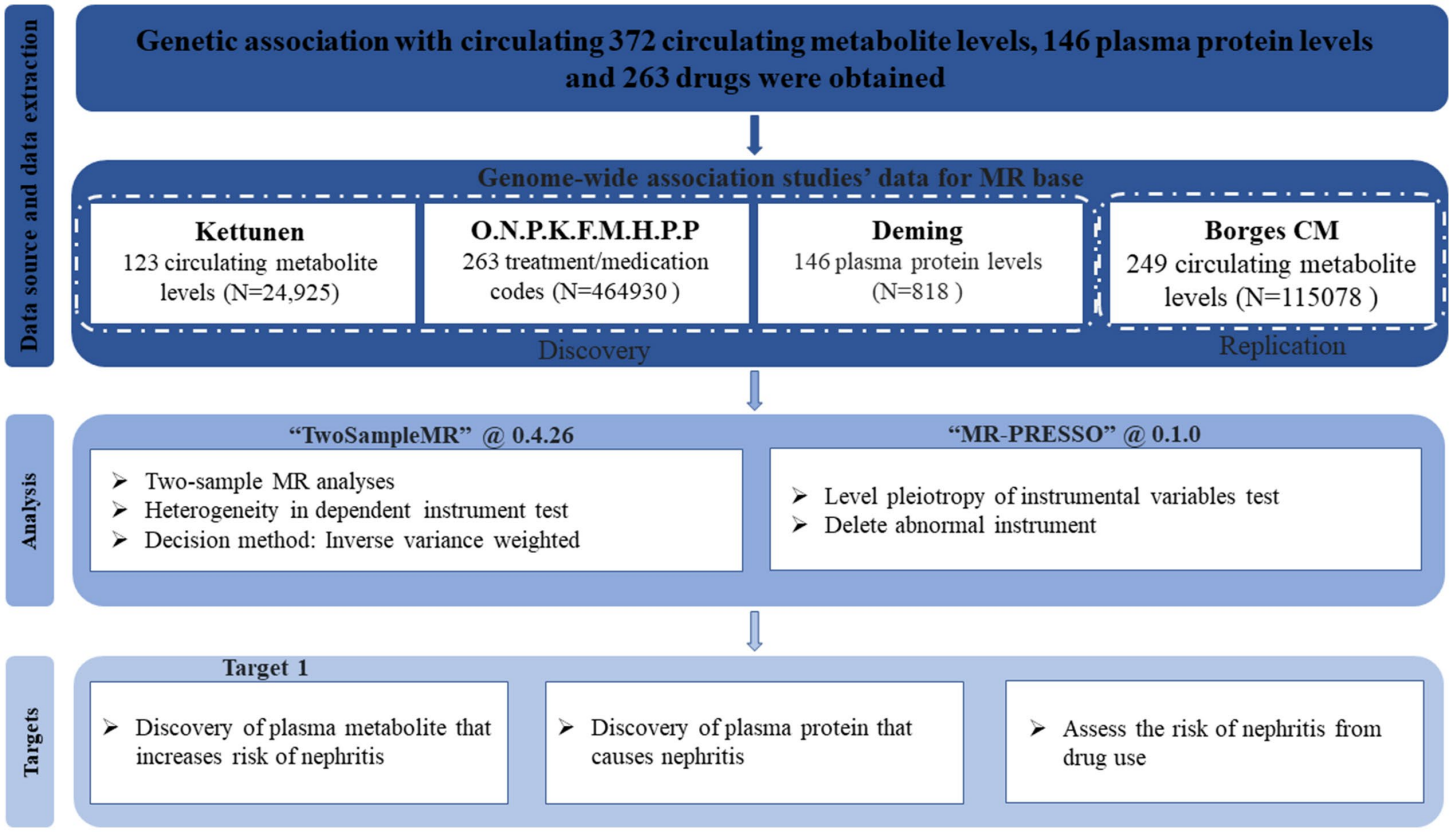
Work process.

The directed acyclic graph (DAG) of the MR part of this study is shown in Figure 1. Summary data for all cohorts used for Mendelian randomization (MR) analysis can be obtained by the “TwoSampleMR” R package, using the keyword “Treatment/medication code” to search for drug treatment cohorts. The GWAS summary statistics used in this two-sample MR survey are the largest to date, and the outcome cohort was extracted from the Finnish cohort in the summary data using the keyword “Nephritis.” The exposure sample cohort uses data from a different source than the outcome GWAS (Finnish cohort) to minimize sample overlap to ensure robustness. Specifically, exposure cohorts of 123 plasma circulating metabolites, plasma proteins, medications, and Omega-3 polyunsaturated fatty acid levels were derived from independent studies (25–30). The drug treatment cohorts of Simvastatin, Atorvastatin, and Multivitamins +/− minerals and the validation cohort of plasma metabolites are also from the British Biobank, which is different from the Finnish database. Detailed descriptions of the cohorts are in Table 1. All these datasets were subjected to Mendelian randomization analysis following the procedures described in Figure 2. Furthermore, kidney-related datasets GSE115857 (IgA nephropathy, 86 samples) and GSE72326 (lupus nephritis, 238 samples) were retrieved from the GEO database to further investigate the lipid metabolism and immunological features of nephritis.

**Table 1:** Data source.

Outcome/Exposure	Author	Year	Case	Control	Total	PMID	Ancestry
Outcome							
Tubulo-interstitial nephritis, not specified as acute or chronic (**TIN**)	Unknown	2018	1,118	201,028	202,146	Unknown	European
Chronic tubulo-interstitial nephritis (**CTIN**)	Unknown	2018	620	201,028	201,648	Unknown	European
Glomerulonephritis (**GN**)	Unknown	2018	4,613	214,179	218,792	Unknown	European
Acute tubulo-interstitial nephritis (**ATIN**)	Unknown	2018	11,216	201,028	212,244	Unknown	European
Exposure							
Plasma circulating metabolites/nephritis	Kettunen	2016			24,925	27,005,778	European
Plasma circulating metabolites/nephritis	Borges CM	2020			115,078	Unknown	European
Plasma protein	Deming	2017			3,146	28,247,064	European
Simvastatin	Ben Elsworth	2018	410,506	52,427	162,933	Unknown	European
Atorvastatin	Ben Elsworth	2018	449,082	13,851	462,933	Unknown	European
Multivitamins +/− minerals	Ben Elsworth	2018	360,727	99,624	460,351	Unknown	European
Fenofibrate	Aslibekyan S	2012			793	23,149,075	European
Serum vitamin D-binding protein levels	Moy KA	2014			1,380	24,740,207	European
Vitamin B6/B12, folic acid	Keene KL	2014			1725	25,147,783	European
Omega-3 polyunsaturated fatty acid	Dorajoo R	2015	644	717	1,361	26,584,805	Singaporean Chinese

### 2.2 Mendelian randomization

The Inverse variance weighting (IVW) method was employed as the primary decision-making approach in all Mendelian randomization (MR) analyses conducted in this study, although a total of five MR methods were used, including Weighted median regression, IVW, Mendelian randomization-Egger (MR Egger), Simple mode, and Weighted mode (31). Because the method can indeed satisfy the causal estimation of instruments with instrumental variable assumptions reported elsewhere (21). Additionally, MR-Egger regression and MR-PRESSO were utilized to assess the potential presence of horizontal pleiotropy among instrumental variables.

### 2.3 Sensitivity analysis

Subsequently, all exposure instrumental variables (IVs) used in the sensitivity analysis showed significant correlation with the exposure in Mendelian randomization (MR) (*p* < 5E-8), ensuring the relevance assumption in MR. To maintain the independence assumption in MR, we selectively chose IVs with linkage disequilibrium (LD) < 0.01 after clustering in 10,000 kb windows. Subsequently, downstream sensitivity analysis was conducted on these IVs that met the first two MR assumptions. The sensitivity analysis employed a penalization approach, implementing a systematic leave-one-out method to assess potential pleiotropy for each single nucleotide polymorphism (SNP) and to test adherence to the exclusion restriction assumption. The random-effect inverse-variance weighted (IVW) method was regarded as the primary analysis (31), and MR-Egger, weighted median, weighted mode, simple mode, and MR-pleiotropy residual sum and outlier (MR-PRESSO) were further employed as complementary methods (32). Additionally, heterogeneity among instrumental variables was assessed using the Q-statistic (*p* < 0.05). MR-Egger regression, along with Mendelian randomization pleiotropy residuals and outliers (MR-PRESSO), was utilized to evaluate horizontal pleiotropy. Evidence of horizontal pleiotropy was based on MR-Egger intercept values significantly deviating from zero (*p* < 0.05). The pleiotropy test assessed the presence of pleiotropic effects, with *p*-values > 0.05 considered indicative of no pleiotropy. Because this article is an “exploratory study,” which means discovering as many potentially positive things as possible, multiple testing correction is not performed. All analyses were performed using the “TwoSampleMR” and “MR-PRESSO” packages in R 4.2.3.

### 2.4 Analysis of lipid metabolism characteristics in IgA nephropathy and lupus nephritis

The “limma” package was used to identify differentially expressed genes between pathological and normal samples (FDR < 0.05, fold change >1.5). Subsequently, lipid metabolism-related genes were obtained from previous studies, which were used to obtain differentially expressed lipid metabolism-related genes. Furthermore, the DAVID tool^1^ (33) was employed for Gene Ontology (GO) and Kyoto Encyclopedia of Genes and Genomes (KEGG) enrichment analysis, and terms with high count values were visualized using the “ggplot2” package to generate bubble plots.

### 2.5 Analysis of immunological characteristics in IgA nephropathy and lupus nephritis

Three immune cell infiltration calculation methods, Cibersort^2^ (34), QuantiSEQ^3^ (35), EPIC^4^ (36), and one immune score cell calculation method, ESTIMATE^5^ (37), were employed to evaluate the immunological characteristics of IgA nephropathy and lupus nephritis. The Kruskal-Wallis test was used to identify differences in immune cell infiltration and immune scores between pathological and control samples (*p* < 0.05).

## 3 Result

### 3.1 Plasma metabolites MR analysis

**Figure 3:**
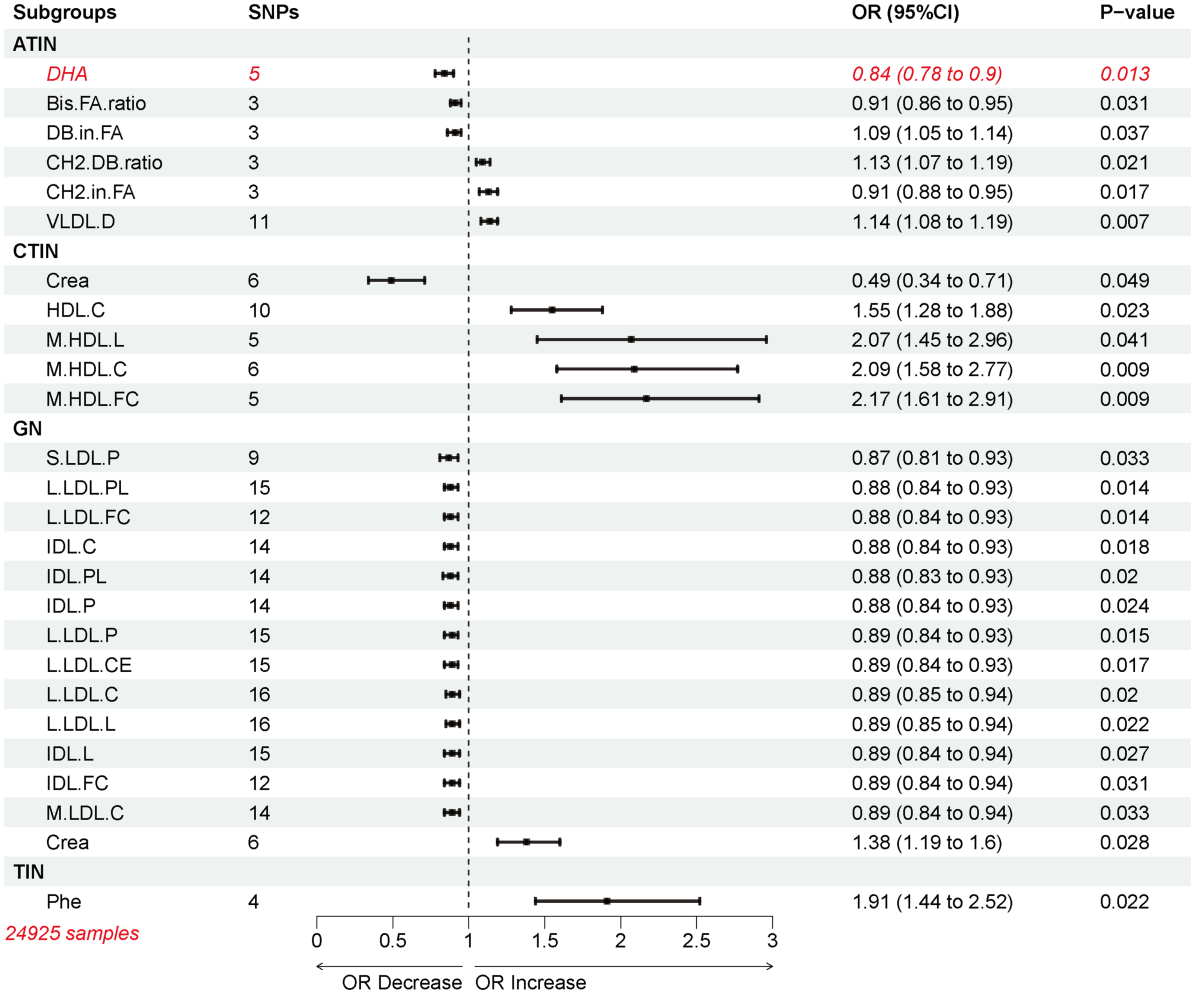
Mendelian randomization of 123 plasma circulating metabolites with nephritis.

In their genome-wide association study, Kettunen et al. investigated 123 circulating metabolites in 24,925 individuals (25), enabling the use of Mendelian randomization (MR) to unveil the widespread causal impact of circulating metabolites on various diseases. Employing the inverse variance weighted (IVW) method as the primary approach in MR analysis, a total of 26 genetically predicted circulating metabolites were found to be associated with the risk of nephritis (Figure 3). Notably, various forms of high-density lipoprotein (HDL), including M.HDL.C (odds ratio (OR): 2.091, 95% confidence interval (CI): 1.204, 3.631), M.HDL.FC (OR: 2.165, 95% CI: 1.211, 3.870), HDL.C (OR: 1.549, 95% CI: 1.062, 2.259), and M.HDL.L (OR: 2.073, 95% CI: 1.032, 4.163), were identified as risk factors for chronic tubulointerstitial nephritis (CTIN). Additionally, phenylalanine (Phe) (OR: 1.906, 95% CI: 1.100, 3.303) was found to be a risk factor for tubulo-interstitial nephritis, not specified as acute or chronic (TIN), while Creatinine (Cre) was identified as a protective factor for CTIN (OR: 0.493, 95% CI: 0.244, 0.996) and a high-risk factor for glomerulonephritis (GN) (OR: 1.378, 95% CI: 1.063, 1.834). It is worth noting that different subtypes of nephritis seemed to correspond to different types of lipoproteins, except for acute tubulointerstitial nephritis (ATIN), where the risk factors were related to fatty acid moieties and proportions (Supplementary Figure S1A). For instance, HDL was identified as a risk factor for CTIN (Supplementary Figure S1B), while LDL and IDL were risk factors for GN (Supplementary Figure S2A). Among the 123 circulating metabolites, only phenylalanine was found to be a risk factor for TIN (Supplementary Figure S2B).

### 3.2 Sensitivity analysis

The MR-PRESSO global heterogeneity test showed no horizontal pleiotropy for all instrumental variables (P Global Test >0.05) (Supplementary Table S1). Cochran Q statistics indicated no heterogeneity in the associations between the 26 circulating metabolic traits and the disease (Supplementary Table S2) (Q pval >0.05). However, using the MR-Egger intercept, all risk factors representing GN, except for Crea, exhibited horizontal pleiotropic effects (Supplementary Table S3).

Single MR analysis revealed CH2.in.FA as the most highly correlated independent risk factor for ATIN (Supplementary Figure S3A). In CTIN, M.HDL.L, M.HDL.FC, M.HDL.C, and Crea showed correlation coefficients exceeding 0.7 (Supplementary Figure S3D). Crea was also the most highly correlated independent risk factor for GN (Supplementary Figure S3B), and Phe exhibited a high independent correlation within TIN (Supplementary Figure S3C).

Leave-one-out test results indicated that prominent risk factors did not have any SNP independently driving the corresponding MR results (Supplementary Figure S4). No evidence of directional pleiotropy was observed in the funnel plots (Supplementary Figure S5). Numerous SNPs exhibited the highest independent correlation with GN risk, including rs2731672, rs4939884, rs6507939, rs6507939, rs9987289, rs1800961, rs4939883, rs9987289, rs1461729, rs2126259, and rs2126259 (B > 1, *p* < 0.05), among others. Additionally, the B coefficient for the rs10265221 instrumental variable representing Crea reached 0.86 (Supplementary Table S4).

### 3.3 Additional database validation

**Figure 4:**
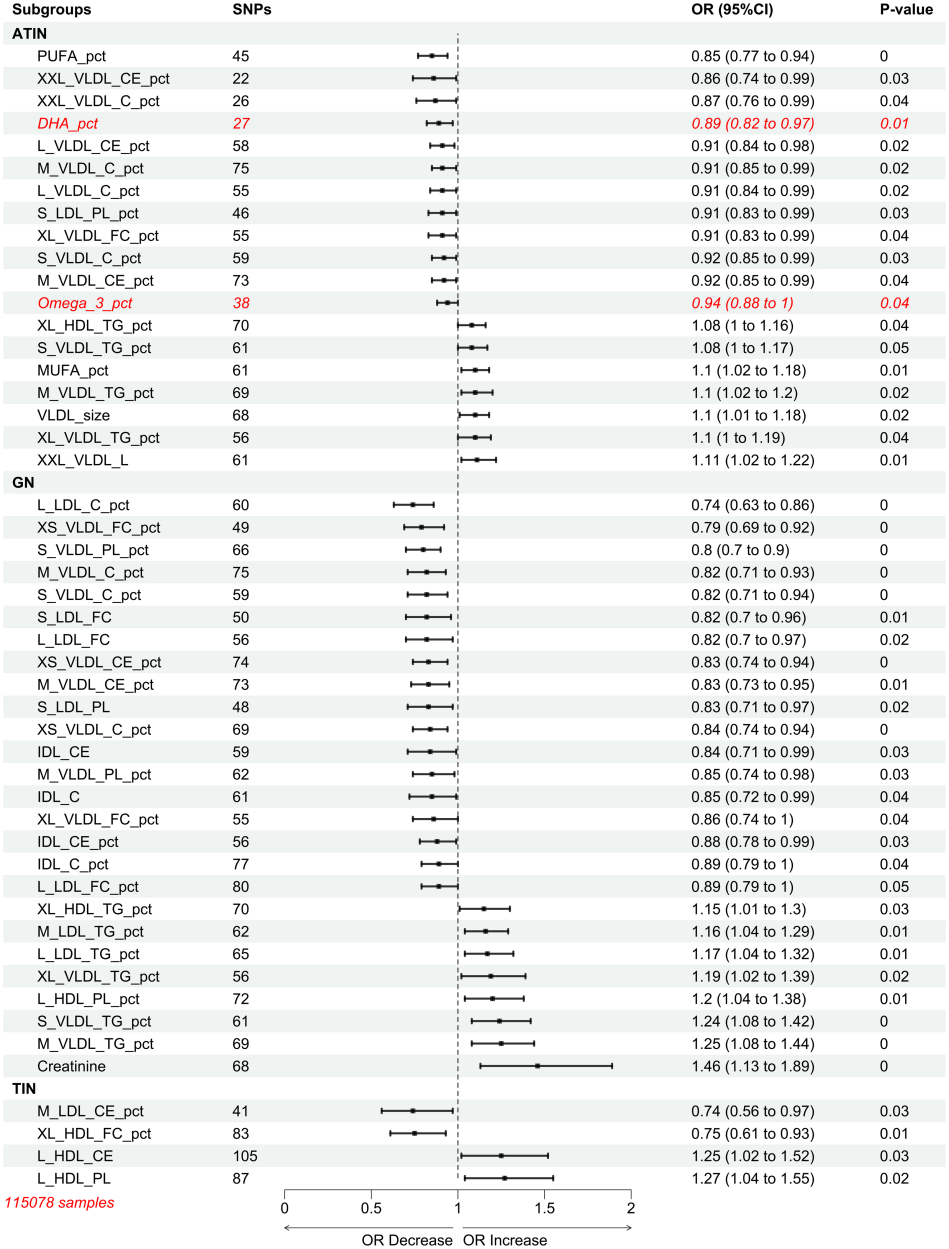
Mendelian randomization results of inverse variance weighted (IVW) estimates of associations between 249 plasma circulating metabolites and nephritis.

We conducted Mendelian randomization studies on a cohort of 115,078 samples from another MR Base. Consistent with the previous results, different types of nephritis appeared to correspond to different types of lipoproteins. Specifically, VLDL, LDL, and IDL were the primary risk factors for GN, while VLDL was the main risk factor for ATIN. Notably, polyunsaturated fatty acids (PUFA) and Docosahexaenic Acid (DHA) were newly discovered protective factors for ATIN. For TIN without specific subtyping, HDL emerged as a more reliable risk factor for TIN compared to LDL (Figure 4).

Based on the study by Deming et al., we also examined the impact of 56 proteins in the plasma of 818 individuals on the outcome of nephritis (27) (Supplementary Table S5). The results revealed only one risk factor, APOH, for TIN (OR = 1.239985889, LOWER1.139454911, UPPER1.34938644). This may be attributed to the limitations of the sample size (818 individuals) and the depth of proteomic sequencing (56 proteins). Overall, these findings collectively suggest that the content of lipoproteins in the plasma is a risk factor for developing nephritis.

### 3.4 Lipid-lowering drugs in nephritis treatment

In independent cohorts, docosahexaenoic acid (DHA), an Omega-3 polyunsaturated fatty acid, consistently emerged as a risk factor for acute tubulointerstitial nephritis (ATIN). Investigating relevant medications within the OMEGA-3 category in the MRbase database becomes essential. Our analysis indicates that reducing lipoprotein-associated phospholipase A2 (Lp-PLA2) via statin therapy can prevent ATIN, while an increase in Lp-PLA2 protects against tubulointerstitial nephritis (TIN). Lowering low-density lipoprotein cholesterol (LDL-C) with statins was deemed preventive against glomerulonephritis (GN) (Supplementary Figure S6A). However, fenofibrate, the second most commonly used lipid-lowering drug, showed no nephritis risk association (Supplementary Table S6).

Excessive niacin and B-complex vitamin supplementation raised nephritis risk. High vitamin B12 levels in plasma increased the likelihood of Chronic Tubulointerstitial Nephritis (CTIN), while elevated vitamin B levels post-ischemic stroke showed the same trend. Interestingly, multivitamin supplementation without appropriate mineral intake increased ATIN risk (Supplementary Figure S6B). Alterations in plasma Omega-3 polyunsaturated fatty acid levels were assessed. Decreased α-Linolenic acid (ALA) percentages in plasma contributed to ATIN, GN, and TIN. Dysregulation of Eicosapentaenoic Acid (EPA) correlated negatively with ATIN risk. Importantly, increased DHA content served as a protective factor for TIN but a risk factor for CTIN (Supplementary Figures S6C–E).

**Figure 5:**
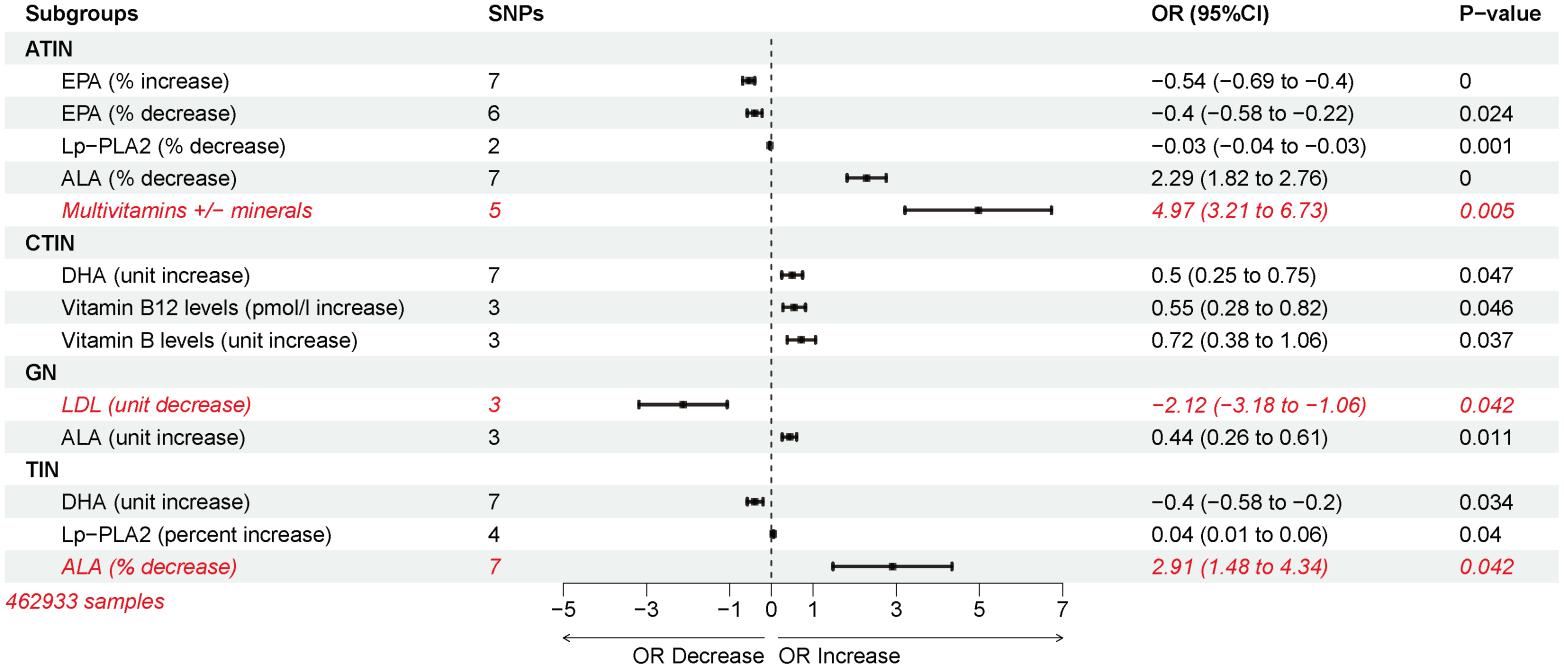
Inverse variance weighting (IVW) estimated risk associations between lipid-lowering medications and changes in plasma omega-3 polyunsaturated fatty acid levels and nephritis.

In summary, among the various factors analyzed, Multivitamins+/minerals-plasma levels emerged as the highest risk factor for ATIN, followed by decreased ALA percentage, also a top risk factor for TIN. Reduction in LDL-C emerged as a protective factor against GN, reducing the significance of other exposure factors (Figure 5).

### 3.5 Potential risks of other medications in nephritis

**Figure 6:**
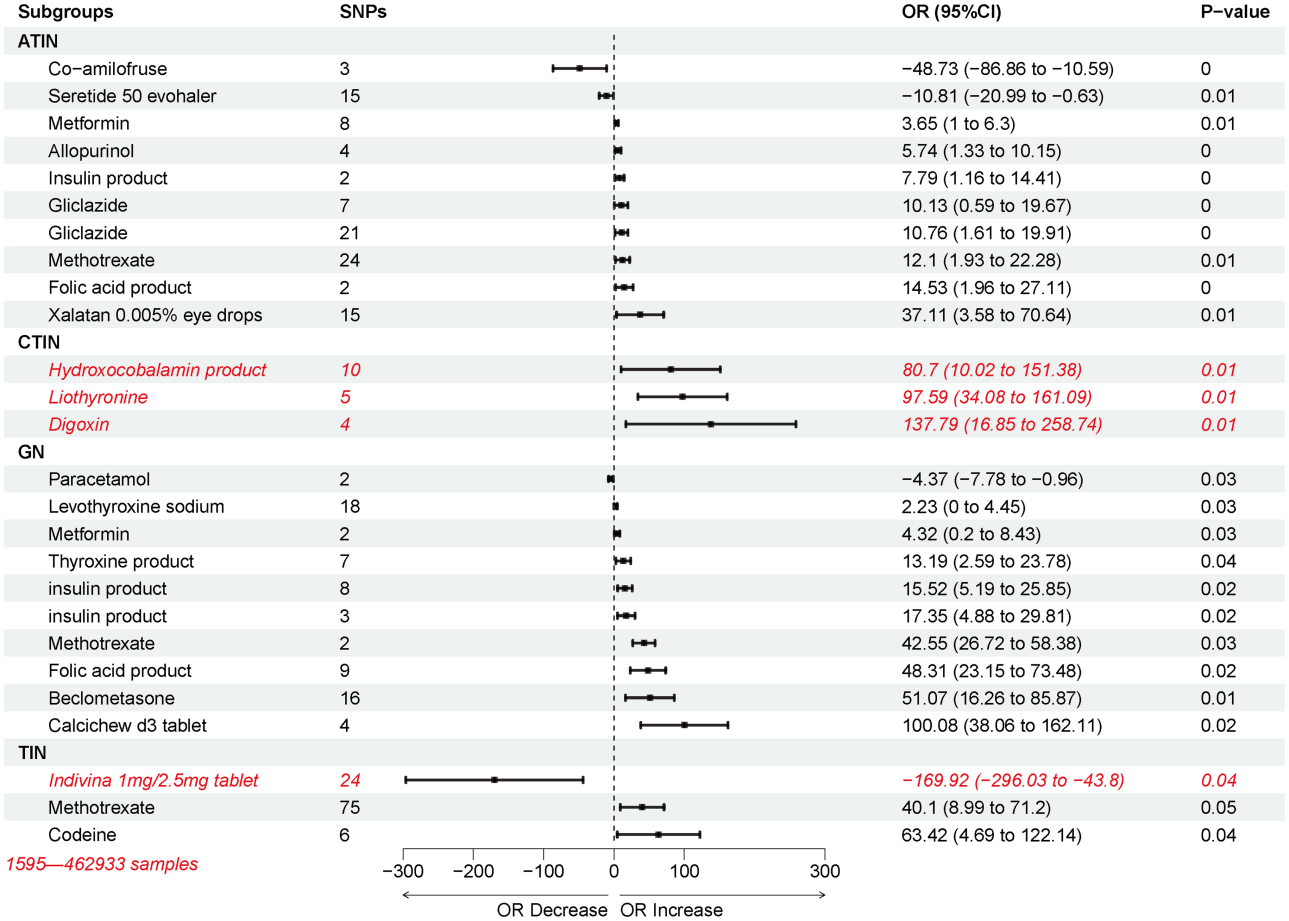
Inverse variance weighting (IVW) estimated the risk association between potential non-nephritic medications and nephritis.

To explore the possibility that other commonly used drugs in clinical practice could trigger nephritis, we extracted genome-wide association data for 263 drugs from the MR base database using the keyword “Treatment/medication code.” Using the IVW method, we assessed the causal impact of these medications on nephritis outcomes. Most of the drug use was associated with an increased likelihood of developing nephritis, with exceptions observed for Co-amilofruse, Seretide 50 evohaler, Paracetamol, and Indivina 1 mg/2.5 mg tablet (Figure 6). Drugs that are causally linked to the risk of nephritis are often used to treat other conditions, such as Thyroid, Diabetes, Respiratory and Vitamin deficiency. Specifically, Methotrexate and Insulin products emerged as potential high-risk factors for various forms of nephritis. Furthermore, the usage of Digoxin was identified as a high-risk factor for CTIN, followed by Hydroxocobalamin product and Liothyronine. In the case of GN, the use of Calcichew d3 tablet was associated with a high-risk profile, followed by Beclometasone and Folic acid product (Figure 6).

### 3.6 Transcriptomics reveals lipid metabolism in kidney diseases

Publicly available datasets from GEO (GSE115857–86 samples, GSE72326–238 samples) were analyzed for IgA nephropathy and lupus nephritis. Differential analysis identified 936 and 1,063 differentially expressed genes for IgA nephropathy and lupus nephritis, respectively. (Supplementary Figures S7A,D).

We assessed 28 and 47 differentially expressed genes related to lipid metabolism in IgA nephropathy and lupus nephritis, respectively, based on our previous research involving 776 lipid metabolism-related genes. Functional enrichment analysis (DAVID 6.8) showed that these genes predominantly localized to the endoplasmic reticulum membrane and were associated with functions like heme binding, iron ion binding, and monooxygenase activity. They played roles in fatty acid metabolic processes, long-chain fatty acid metabolic processes, Metabolic pathways, the PPAR signaling pathway, and insulin resistance (Supplementary Figure S7G).

Abnormal lipid metabolism pathways in lupus nephritis mirrored those in IgA nephropathy. Differentially expressed molecules were primarily located at the endoplasmic reticulum membrane, engaged in lipid metabolic processes, and exhibited oxidoreductase activity (involving the CH-CH group of donors). These genes were linked to Metabolic pathways, Glycerophospholipid metabolism, and Fatty acid metabolism pathways (Supplementary Figure S7H).

### 3.7 Immune infiltration characteristics of IgA nephritis and lupus nephritis

In recent years, immune checkpoint inhibitors (ICIs) have been effective in cancer treatment and managing kidney diseases, including nephritis induced by immune deposits (38, 39). New algorithms and “next-generation” sequencing enable computational assessment of immune infiltration differences between nephritis and healthy kidney tissue (34–37). Using CIBERSORT, EPIC, QUANTISEQ, and ESTIMATE methods, immune infiltration disparities in IgA nephropathy and lupus nephritis were revealed. Both conditions showed abnormal B cell and CD4-positive T cell infiltration (Supplementary Figures S8A,B), consistently observed with other algorithms (Supplementary Figures S8C–F). IgA nephropathy exhibited abnormal infiltration of NK cells, monocytes, and activated dendritic cells (Supplementary Figures S8A,C,E), while lupus nephritis displayed abnormal Treg cells, M0 macrophages, and M1 macrophages infiltration (Supplementary Figures S8B,D,F).

## 4 Discussion

In this study, we utilized a two-sample Mendelian randomization (MR) approach to investigate causal connections between plasma metabolites and nephritis. Bioinformatics analysis of transcriptomics data revealed distinct lipid metabolism and immune traits in IgA nephropathy and lupus nephritis.

The MR analysis of plasma circulating metabolites in two cohorts, Kettunen (24,925 samples) and Borges CM (115,078 samples), demonstrated that different types of lipoproteins are causally associated with distinct types of nephritis. Specifically, HDL-related circulating metabolites showed a tendency to be causally associated with CTIN, consistent with previous reports (40, 41). While randomized controlled trials did not show evident causal relationships between LDL and IDL in GN, reports indicated a negative correlation between LDL-C and renal function as well as kidney disease (42, 43). Unfortunately, current evidence does not support a causal relationship between VLDL-related circulating metabolites and ATIN. Lp-PLA2 is one of the isoforms in the phospholipase superfamily and is secreted into plasma by macrophages, T cells and mast cells (44). Lp-PLA is currently a recognized marker of cardiovascular disease, but it plays different roles in inflammation and atherosclerosis depending on the lipoprotein bound in plasma (45). The results of this study show that the decrease in Lp-PLA2 caused by statins is a protective factor for ATIN, and the increase in Lp-PLA2 caused by statins is also a protective factor for TIN. This may be caused by intra-group differences in TIN (Tubulo-interstitial nephritis, not specified as acute or chronic), because the summary data we obtained failed to distinguish ATIN and TIN in the TIN group. In addition, the clinical manifestations of TIN with different causes will be different (46–49), and different types of nephritis will also have different pathological characteristics and clinical outcomes (50–53). Therefore, compared with TIN, the conclusion that decreased Lp-PLA2 is a protective factor for ATIN is more reliable, while the conclusion that increased Lp-PLA2 is a protective factor for TIN needs to be treated with caution.

In the mentioned cohorts, an intriguing negative link between DHA and ATIN led to deeper exploration of Omega-3 fatty acid drugs (e.g., statins, niacin, fenofibrate) in nephritis. Surprisingly, multivitamin supplementation without minerals was tied to higher ATIN risk, warranting further investigation. Statins, commonly employed in CKD treatment for reducing cardiovascular disease risk (54), showed interesting associations in our study. Specifically, statin-induced ALA reduction was found to increase the risk of ATIN and GN, while statin-induced LDL reduction exhibited a protective effect against GN. Previous studies have identified ALA as a predictive biomarker for chronic kidney disease (CKD) (55), although its ability to slow the progression of IgA nephropathy remains inconclusive (56). In contrast, LDL has been suggested to play a role in CKD progression in certain observational and mechanistic studies (42, 57), and some reports even propose lower LDL levels to enhance the therapeutic benefits of statins in treating patients with chronic kidney disease (58). Based on these findings, we recommend cautious ALA supplementation with statins in CKD for optimized efficacy.

In recent years, numerous drugs have been discovered to possess potential for treating other diseases (59, 60), a phenomenon termed “new uses of old drugs” (61). Therefore, driven by curiosity about whether drugs used for other diseases are beneficial in treating nephritis, we further explored the causal relationship between 263 other drugs and nephritis. Among the 26 positive results, digoxin, hydroxocobalamin, and liothyronine were identified as the only three high-risk factors for CTIN. Among these, Digoxin is commonly believed to reduce the risk of CKD through its treatment of cardiovascular diseases (62, 63). However, our research results provide support for the notion that excessive use of Digoxin is, in fact, one of the high-risk factors for CKD (64). Additionally, thyroid hormones regulated by liothyronine and thyroid diseases are often closely related to CKD (65–67). In an MR study concerning drugs, concluding without considering hidden co-founders and pathway analysis is risky. Therefore, the findings of these drugs must be treated with caution. They can only serve as a basis for subsequent research and cannot be directly used for clinical recommendations and guidance.

The aforementioned findings indicate that lipoproteins and fatty acids in circulating metabolites are potential risk factors for nephritis, a conclusion supported by proteomic data analysis (68, 69). For instance, Chen et al. conducted quantitative proteomic analysis on 59 IgA nephropathy cases and 19 normal controls, identifying novel molecular subtypes (70). They highlighted specific alterations in fatty acid β-oxidation, the TCA cycle, glycolysis, and oxidative phosphorylation among these subtypes, findings consistent with those of Dong et al. (71). The gut microbiome, proteomic and metabolomic landscape of renal diseases offers insights into pathogenesis, treatment, and prevention, yet transcriptomic studies remain scarce (50, 72–75). Scientists are also promoting the discovery of new treatments and targets for various types of nephritis (76–79). Indeed, integrating transcriptomics with other omics can further advance the discovery of novel mechanisms and biomarkers for renal diseases (80). Therefore, we leveraged transcriptomic data from IgA nephropathy and lupus nephritis to elucidate their lipid metabolism and immune features, as dysregulation of immune regulatory molecules or immune function is also considered a contributing factor to nephritis onset and progression (38). Dysregulation of lipid oxidation, uptake, and biosynthesis is deemed a predisposing factor for CKD, a notion supported by an early pioneering study (9, 81). Additionally, IgA nephropathy and lupus nephritis exhibit aberrant B-cell and CD4 T-cell infiltration. Mechanistic and observational studies suggest that modulation through B cells and CD4 T cells can effectively suppress the development of IgA nephropathy and lupus nephritis, findings consistent with our study results (82–87).

Compared to other Mendelian randomization studies on kidney diseases, the strength of our research lies in the direct use of clinically diagnosed outcomes as the outcome variables, including Acute tubulo-interstitial nephritis, Chronic tubulo-interstitial nephritis, Glomerulonephritis, and Tubulo-interstitial nephritis. Instead of relying on traditional measures of kidney function such as glomerular filtration rate or creatinine clearance, we have utilized these clinically relevant diagnostic outcomes, enabling a clearer delineation of the causal relationships between exposure factors and diseases. Moreover, the robust sensitivity analysis ensures the reliability of the instrumental variables, as described in the Methods section.

### 4.1 Study limitations

This study used summary statistics data from MR base, limiting our ability to explore U-shaped relationships between exposure factors and nephritis. The availability of larger sample sizes was restricted, affecting statistical power for some instrumental variables. In addition, in order to explore more potential positive exposure factors, this study did not correct the results, which may lead to false positives. Not only that, when analyzing the causal association between exposure factors (Omega-3 polyunsaturated fatty acid) and nephritis outcomes, a cohort of European ancestry was not available, and the different ancestry of the exposure and outcome samples may lead to biased results. Although this result meets the criteria for a test of heterogeneity, this result needs to be interpreted with caution. Moreover, due to the inability to obtain complete original data, bidirectional causal MR analysis was not performed, and it was also impossible to further clarify the pathological stratification of outcome variables to obtain more valuable clinical significance. Nevertheless, the study identified the studied exposure factors as causal factors for nephritis.

## 5 Conclusion

Our MR study supports a causal link between plasma metabolites, especially DHA, and nephritis. Lipid-lowering drugs offer potential as nephritis treatments, possibly in combination with other medications for enhanced outcomes. Non-nephritic drugs like Indivina and co-amilofruse show promise for TIN treatment. However, caution is needed with drugs like Digoxin, hydroxocobalamin, and liothyronine, depending on the patient’s renal function. Additionally, transcriptomics data analysis confirms abnormal lipid metabolism and immune features in nephritis, advancing our understanding of its pathogenesis and potential for targeted therapies.

## Data availability statement

The data presented in the study are deposited in the GEO repository, accession number GSE115857 (https://www.ncbi.nlm.nih.gov/geo/query/acc.cgi?acc=GSE115857) and GSE72326 (https://www.ncbi.nlm.nih.gov/geo/query/acc.cgi?acc=GSE72326).

## Ethics statement

Ethical approval was not required for the study involving humans in accordance with the local legislation and institutional requirements. Written informed consent to participate in this study was not required from the participants or the participants’ legal guardians/next of kin in accordance with the national legislation and the institutional requirements.

## Author contributions

FS: Conceptualization, Data curation, Formal analysis, Methodology, Writing – original draft. YY: Conceptualization, Data curation, Investigation, Software, Writing – original draft. DW: Supervision, Validation, Writing – original draft. RW: Methodology, Supervision, Writing – original draft. RL: Validation, Writing – original draft. YZ: Methodology, Writing – original draft. EL: Data curation, Writing – original draft. MW: Writing – original draft. YT: Resources, Supervision, Writing – review & editing. YD: Investigation, Resources, Supervision, Writing – review & editing. YX: Funding acquisition, Project administration, Supervision, Writing – review & editing.

## Footnotes

^1^https://david.ncifcrf.gov/tools.jsp

^2^https://cibersortx.stanford.edu/

^3^https://icbi.i-med.ac.at/software/quantiseq/doc/

^4^http://epic.gfellerlab.org

^5^https://sourceforge.net/projects/estimateproject/
